# Predictive role of intracranial PD-L1 expression in a real-world cohort of NSCLC patients treated with immune checkpoint inhibition following brain metastasis resection

**DOI:** 10.1007/s11060-024-04590-w

**Published:** 2024-02-15

**Authors:** David Wasilewski, Julia Onken, Paul Höricke, Jan Bukatz, Selin Murad, Anton Früh, Zoe Shaked, Martin Misch, Anja Kühl, Oliver Klein, Felix Ehret, David Kaul, Helena Radbruch, David Capper, Peter Vajkoczy, David Horst, Nikolaj Frost, Philip Bischoff

**Affiliations:** 1 Department of Neurosurgery, Charité – Universitätsmedizin Berlin, Corporate Member of Freie Universität Berlin and Humboldt-Universität Zu Berlin, Berlin, Germany; 2 Charité Comprehensive Cancer Center, Charité – Universitätsmedizin Berlin, Corporate Member of Freie Universität Berlin and Humboldt-Universität Zu Berlin, Berlin, Germany; 3 German Cancer Consortium (DKTK), Partner Site Berlin, and German Cancer Research Center (DKFZ), Heidelberg, Germany; 4 Berlin Institute of Health (BIH) Charité, Berlin Institute of Health at Charité – Universitätsmedizin Berlin, Charitéplatz 1, 10117 Berlin, Germany; 5 Institute of Pathology, Charité – Universitätsmedizin Berlin, Corporate Member of Freie Universität Berlin and Humboldt-Universität Zu Berlin, Berlin, Germany; 6 Department of Radiation Oncology, Charité – Universitätsmedizin Berlin, Corporate Member of Freie Universität Berlin and Humboldt-Universität Zu Berlin, Berlin, Germany; 7 Institute of Neuropathology, Charité – Universitätsmedizin Berlin, Corporate Member of Freie Universität Berlin and Humboldt-Universität Zu Berlin, Berlin, Germany; 8 Department of Infectious Diseases and Pulmonary Medicine, Charité – Universitätsmedizin Berlin, Corporate Member of Freie Universität Berlin and Humboldt-Universität Zu Berlin, Berlin, Germany

**Keywords:** Brain metastasis, NSCLC, PD-L1, Discordance, Survival, PFS

## Abstract

**Background:**

Emerging evidence suggests that treatment of NSCLC brain metastases with immune checkpoint inhibitors (ICIs) is associated with response rates similar to those of extracranial disease. Programmed death-ligand 1 (PD-L1) tumor proportion score (TPS) serves as a predictive biomarker for ICI response. However, the predictive value of brain metastasis-specific (intracranial) PD-L1 TPS is not established. We investigated the role of intra- and extracranial PD-L1 TPS in NSCLC patients treated with ICI following brain metastasis resection.

**Methods:**

Clinical data from NSCLC patients treated with ICI following brain metastasis resection (*n* = 64) were analyzed. PD-L1 TPS of brain metastases (*n* = 64) and available matched extracranial tumor tissue (*n* = 44) were assessed via immunohistochemistry. Statistical analyses included cut point estimation via maximally selected rank statistics, Kaplan–Meier estimates, and multivariable Cox regression analysis for intracranial progression-free survival (icPFS), extracranial progression-free survival (ecPFS), and overall survival (OS).

**Results:**

PD-L1 expression was found in 54.7% of brain metastases and 68.2% of extracranial tumor tissues, with a median intra- and extracranial PD-L1 TPS of 7.5% (0 – 50%, IQR) and 15.0% (0 – 80%, IQR), respectively. In matched tissue samples, extracranial PD-L1 TPS was significantly higher than intracranial PD-L1 TPS (*p* = 0.013). Optimal cut points for intracranial and extracranial PD-L1 TPS varied according to outcome parameter assessed. Notably, patients with a high intracranial PD-L1 TPS (> 40%) exhibited significantly longer icPFS as compared to patients with a low intracranial PD-L1 TPS (≤ 40%). The cut point of 40% for intracranial PD-L1 TPS was independently associated with OS, icPFS and ecPFS in multivariable analyses.

**Conclusion:**

Our study highlights the potential role of intracranial PD-L1 TPS in NSCLC, which could be used to predict ICI response in cases where extracranial tissue is not available for PD-L1 assessment as well as to specifically predict intracranial response.

**Supplementary Information:**

The online version contains supplementary material available at 10.1007/s11060-024-04590-w.

## Introduction

Brain metastases are common in patients with solid malignancies, with up to 50% of patients developing brain metastases during the course of their disease, and negatively impact clinical outcomes [1, 2]. Local treatment including radiation therapy (RTx) or stereotactic radiosurgery (SRS), with or without microsurgical resection can improve local (intracranial) control [1–4]. In contrast, systemic therapies including immune checkpoint inhibitors (ICIs) improve overall survival (OS) in brain metastasis patients, mainly mediated via extracranial disease control and thus prolonging extracranial progression-free survival (ecPFS) [5–7]. Treatment with ICIs, which are directed against the immune checkpoint programmed cell death ligand 1 (PD-L1), demonstrated a marked OS improvement in patients with melanoma and non-small cell lung cancer (NSCLC) as well as other entities [8–11]. In contrast, the impact of ICI treatment on intracranial progression-free survival (icPFS) is still ill-defined as patients with brain metastases have been frequently excluded from clinical trials [12–14].

PD-L1 is a transmembrane immunoregulatory protein expressed on tumor and antigen-presenting cells. Interaction of tumor cell-derived PD-L1 and programmed-death 1 (PD-1) on T cells can elicit tumor immune escape by negative regulation of T cells. Higher PD-L1 tumor proportion scores (TPS) as well as expression of other markers such as PD-1, presence of tumor-infiltrating lymphocytes (TILs) or combinations of immunohistochemical staining such as PD-L1 in combination with CD8 + T cells represent surrogate markers of an immune-active or “hot” tumor immune microenvironment (TME) and correlate with objective response rates of tumors treated with antibodies directed against the PD-L1/PD-1 axis [15–17].

However, quantification of PD-L1 TPS by immunohistochemistry (IHC) is nowadays the only tissue-based biomarker that is used in the clinical routine to predict ICI response and serves for patient selection [18–20]. In this regard, it has been argued that the TME in brain metastases may differ substantially from matched extracranial tumor tissue [21–24].

The role of PD-L1 in predicting response and outcome of ICI-treated patients with brain metastasis remains elusive and has not been assessed in a comprehensive manner. In that regard, studies comparing the predictive relevance of brain metastasis-specific (intracranial) and extracranial PD-L1 TPS are lacking. Hence, it is unknown whether PD-L1 assessment in brain metastases might provide additional information to guide patient selection for ICI therapy. We aimed to analyze the role of intra- and extracranial PD-L1 TPS specifically in NSCLC patients who received ICIs after neurosurgical brain metastasis resection and postoperative RTx.

## Methods

### Patient cohort

Patients with histologically proven NSCLC either based on resected or biopsied tissue originating from primary, extracranial metastatic lesion or lymph node metastasis as well as histologically confirmed NSCLC brain metastasis during their disease between January 2016 and April 2023 were included in this retrospective study (Table 1, Supplementary figure 1). All patients underwent brain metastasis resection and were treated inpatient and outpatient at the Department of Neurosurgery and Department of Thoracic Oncology at one of the three sites of the University Medical Center of the Charité. Most patients had synchronous brain metastases at the time of diagnosis and were treatment-naïve at the time of brain metastasis resection, whereas 10 patients had metachronous brain metastasis and received systemic pre-treatment before neurosurgical brain metastasis resection (Table 1). Clinical data, data from imaging studies, histopathological and treatment-related characteristics were retrieved from electronic patient records. Baseline characteristics including graded prognostic assessment (GPA) and Karnofsky performance score (KPS) are summarized and potential confounding factors of intracranial PD-L1 expression including systemic pre-treatment with CTx or ICI or daily treatment with dexamethasone before brain metastasis resection are listed in Table 1. Twenty patients have had no extracranial PD-L1 TPS assessment as these patients did not undergo sampling of extracranial tissue. Missing values were found in the following variables: two missing values for intracranial Ki67, 33 missing values for extracranial Ki67. For six patients daily dexamethasone doses were incomplete. Evaluation of extra- and intracranial disease progression was based on reports from board-certified radiologists. Tumor volumes of the resected lesion were quantified using a semi-automated 3D rendering algorithm in iPlannet (Brainlab, Munich, Germany) using the SmartBrush tool. As for CT staging and MRI follow-up, radiologic tumor assessment was analyzed for treatment efficacy with response evaluation following the discretion of the treating physician according to standardized response evaluation criteria in solid tumours (RECIST) assessment (version 1.1) and immunotherapy response assessment in neuro-oncology (iRANO) criteria (complete response (CR), partial response (PR), stable disease (SD), or progressive disease (PD)) based on retrospective chart reviews without central confirmation. Progression was categorized as either extracranial progression-free survival (ecPFS) or intracranial progression-free survival (icPFS) and separate extracranial and intracranial PFS estimates were calculated. Median time of follow-up was estimated using the reverse Kaplan–Meier method. All outcome parameters including overall OS, icPFS and ecPFS were defined as the time from neurosurgical brain metastasis resection until death or censoring at last known time alive.

**Table 1:** Patient characteristics of the total cohort of resected NSCLC brain metastasis patients. Summary of patient features of the study cohort (*n* = 64), in which all patients were treated with RTx + ICI after brain metastasis resection. All characteristics are given at the time of first brain metastasis resection (baseline), if not stated otherwise. Cumulative daily pre-operative dexamethasone (in mg) equals the sum of daily dexamethasone doses before brain metastasis operation from 13 days before operation (day-13) until one day before operation (day-1). Patients were characterized according to UICC stage (based on the 8th UICC/AJCC TNM edition) and clinical performance including KPS and prognostic score GPA. KPS and GPA were arbitrarily dichotomized into “good” and “bad” according to previous studies in the field [5]. Line of therapy: first line (1L), second line (2L)

Characteristic	*N* = 64
Age, median (IQR)	64.3 (58.1 – 72.1)
Sex, *n* (%)	
Female	27 (42.2%)
Male	37 (57.8%)
Histological subtypes, *n* (%)	
Adenocarcinoma	54 (84.4%)
Pulmonary sarcomatoid carcinoma	1 (1.6%)
Squamous cell cancer	7 (10.9%)
Undifferentiated	2 (3.1%)
UICC at time of NSCLC diagnosis, *n* (%)	
IA	1 (1.6%)
IB	2 (3.1%)
IIIB	1 (1.6%)
IIIC	1 (1.6%)
IV	59 (92.2%)
GPA score, *n* (%)	
Bad GPA score (GPA ≤ 2)	33 (51.6%)
Good GPA score (GPA > 2)	31 (48.4%)
KPS after operation, *n* (%)	
Bad KPS (< 70%)	16 (25.0%)
Good KPS (≥ 70%)	48 (75.0%)
Extracranial metastasis burden, *n* (%)	
No presence of extracranial metastases	37 (57.8%)
Presence of extracranial metastases	27 (42.2%)
Intracranial metastasis burden, *n* (%)	
1 brain metastasis	15 (23.4%)
≥ 2 brain metastases	49 (76.6%)
Tumor volume (mL), median (IQR)	9.6 (5.8 – 20.4)
Edema volume (mL), median (IQR)	94.2 (61.6 – 153.3)
Systemic therapy before brain metastasis resection, *n* (%)	
Naive before brain metastasis resection	54 (84.4%)
Pre-treated before brain metastasis resection	10 (15.6%)
Specific systemic therapy before brain metastasis resection, *n* (%)	
Carboplatin and Nab-Paclitaxel	2 (20.0%)
Carboplatin, Pemetrexed, and Pembrolizumab	1 (10.0%)
Carboplatin, Nab-Paclitaxel and Bevacicumab	1 (10.0%)
Carboplatin, Nab-Paclitaxel and Pembrolizumab	1 (10.0%)
Cisplatin and Gemcitabine	1 (10.0%)
Cisplatin and Pemetrexed (1L), Docetaxel and Seribantumab (2L)	1 (10.0%)
Cisplatin and Vinorelbine	1 (10.0%)
Pembrolizumab	2 (20.0%)
Primary tumor resection, *n* (%)	
No primary tumor resection	49 (76.6%)
Primary tumor resection before brain metastasis resection	7 (10.9%)
Primary tumor resection after brain metastasis resection	8 (12.5%)
Mode of adjuvant radiation therapy, *n* (%)	
SRS	44 (68.8%)
WBRT	20 (31.3%)
Dose of postoperative radiation in Gy, median (IQR)	36 (30.0 – 41.8)
Cumulative pre-operative dexamethasone, median (IQR)	44.0 (0.0 – 120.0)
Cumulative pre-operative dexamethasone dose dichotomized, *n* (%)	
> 52 mg	29 (45.3%)
≤ 52 mg	35 (54.7%)
Systemic therapy after brain metastasis resection, *n* (%)	
ICI + CTx (followed by ICI maintenance therapy)	39 (60.9%)
ICI monotherapy	25 (39.1%)
Specific systemic therapy after brain metastasis resection, *n* (%)	
Carboplatin, Etoposide and Atezolizumab, followed by Atezolizumab maintenance therapy	1 (1.6%)
Carboplatin, Nab-Paclitaxel and Atezolizumab, followed by Atezolizumab maintenance therapy	4 (6.3%)
Carboplatin, Nab-Paclitaxel and Pembrolizumab, followed by Pembrolizumab maintenance therapy	1 (1.6%)
Carboplatin, Nab-Paclitaxel, Nivolumab and Ipilimumab, followed by Nivolumab and Ipilimumab maintenance therapy	3 (4.7%)
Carboplatin, Nab-Paclitaxel, Pembrolizumab, followed by Pembrolizumab maintenance therapy	3 (4.7%)
Carboplatin, Pemetrexed and Pembrolizumab, followed by Pembrolizumab maintenance therapy	18 (28.1%)
Cisplatin, Pemetrexed and Pembrolizumab, followed by Pembrolizumab maintenance therapy	9 (14.1%)
Atezolizumab monotherapy	4 (6.3%)
Nivolumab monotherapy	2 (3.1%)
Pembrolizumab monotherapy	19 (29.7%)

### Immunohistochemical analysis

Specimens from resected (intracranial) brain metastasis tissue (*n* = 64) and available matched extracranial tumor tissue (*n* = 44, comprising 9 resected primary lung tumors, 9 lung biopsies and 26 lymph node biopsies) were considered for analysis. Each case was reviewed by a pathologist or neuropathologist. FFPE tissue sections of 2–3 µm thickness were used for hematoxylin and eosin (H&E) staining and immunohistochemistry (IHC). IHC staining for PD-L1 was performed using a Leica Bond immunostainer (Leica Biosystems) according to manufacturer’s protocols. Briefly, tissue sections were deparaffinized, rehydrated, and heat-induced antigen retrieval was performed by incubation in CC1 mild buffer (Ventana Medical Systems) for 30 min at 100 °C. Subsequently, tissue sections were incubated with the primary monoclonal antibody rabbit anti-PD-L1 (Clone E1L3N, Cell Signaling, #13684) at 1:200 for 60 min followed by incubation with HRP-conjugated secondary antibody (Leica Biosystems) for 32 min, DAB incubation and counterstaining of nuclei with hematoxylin. Tonsil served as positive control. PD-L1 expression was quantified by assessing the tumor proportion score (TPS), i.e. assessing the percentage of tumor cells with positive membranous staining relative to all vital tumor cells. Only cases with at least 100 evaluable tumor cells were included [25].

### Statistical analysis

Descriptive statistics were performed to summarize the presented patient cohort and associated clinical, histopathological, radiological, and treatment-related patient features. Data collection was done with Excel version 14.3.9 (Microsoft). We used R version 1.1.442 (R Foundation) to compute statistics, including frequencies, means, and SDs, to characterize the cohort. For patient baseline characteristics, continuous data were compared across cohorts using the Wilcoxon rank-sum test, while categorical data were compared using Fisher exact or χ2 tests. We used the gtsummary package (version 1.7.2, R Foundation) to describe tabular data of the patient cohort, including categorical and numerical variables. Optimal cut points of PD-L1 TPS for icPFS, ecPFS and OS were determined using the maxstat package (version 0.7–25) displaying maximally selected rank statistics (M) and p-values as well as distribution of optimal cut point values for a given predictor [26]. Median icPFS, ecPFS and OS was estimated by Kaplan–Meier analysis with 95% CI bands being displayed in light color; and log-rank test was used to compare OS and PFS between patient groups; plotting was performed using the survminer package (version 0.4.3, R Foundation). Multivariable Cox regression model for OS, ecPFS and icPFS were computed, including only complete cases, using clinical covariates assumed to be associated with the respective outcomes. Further R packages used for analysis included dplyr (version 1.1.4.), tidyverse (version 2.0.0), and swimplot (version 1.2.0). A p-value < 0.05 was considered significant with p-values being 2-sided. R code and raw data will be made available to researchers on request.

## Results

### Patient characteristics

**Fig. 1:**
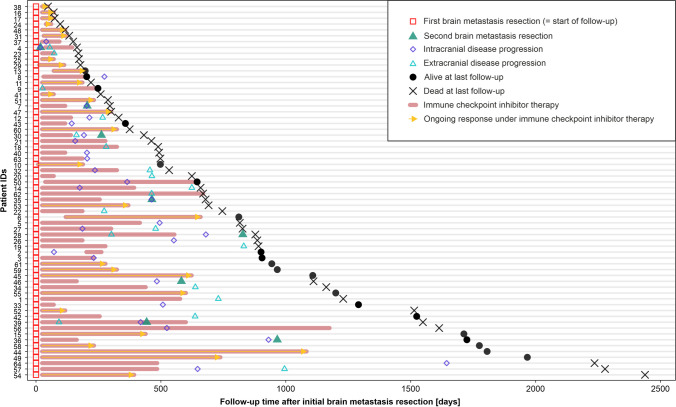
Clinical course of NSCLC brain metastasis patients treated with ICIs after brain metastasis resection. Visualization of the clinical course and administered treatments after primary brain metastasis resection. Follow-up time starts at first brain metastasis resection, indicated by a red square. If applicable, secondary brain metastasis resection is indicated by a green triangle. The time under ICI treatment is marked with a light red bar, including ICI monotherapy, ICI + CTx and ICI maintenance therapy. Ongoing treatment response during ICI treatment is indicated by an orange arrow. Further indicated events include intracranial disease progression (iDP) and extracranial diseases progression (eDP), indicated by an empty diamond or triangle, respectively. Patient status at the time of last follow-up is indicated by a black circle as alive, or black cross as dead. Forty-four patients died during the observation time

Sixty-four patients who underwent resection of NSCLC brain metastases were included in the study (Supplementary figure 1). Baseline was defined as the time of first brain metastasis resection. Patient characteristics at baseline are summarized in Table 1. Median follow-up time from first brain metastasis resection was 57.1 months [95% CI: 40.0—NA]. Fifty-four patients (84.4%) were diagnosed at UICC stage IV with synchronous brain metastasis, therefore had been treatment-naïve before brain metastasis resection. Ten patients suffered from metachronous brain metastasis and have had systemic pre-treatment with first-line systemic treatment in a palliative (8 patients) or curative (2 patients) setting including platin-based CTx, with two of these patients receiving CTx + ICI and two patients receiving ICI monotherapy before brain metastasis resection. Only one of the pre-treated patients received both first line (1L) and second line (2L) systemic pre-treatment before brain metastasis resection. Two patients within the group of pre-treated patients received a brain metastasis-specific local treatment including SRS before brain metastasis resection in addition to systemic treatment. After brain metastasis resection, 39 patients received a combination of ICI and platin-based CTx followed by maintenance ICI, whereas 25 patients received ICI monotherapy (Table 1). ICI treatment was administered for a median number of 9 cycles (95% CI: 6 – 13) and mean duration of 207 days (95% CI: 138 – 276). At time of brain metastasis resection, 27 patients (42.2%) had additional extracranial organ metastases, whereas 37 patients (57.8%) showed no sign of extracranial metastasis. 49 patients (76.6%) had two or more brain metastases. Median cumulative daily dexamethasone dose during a time period of 13 days before operation was 44.0 mg (0.0 – 120.0, IQR). There was no correlation between intracranial PD-L1 TPS and cumulative pre-operative dexamethasone, or other clinical parameters (Supplementary figure 2). The clinical course of included patients is displayed in Fig. 1. All 64 patients of the study cohort receiving postoperative RTx + ICI showed better OS, ecPFS and icPFS compared to patients undergoing other postoperative therapies after brain metastasis resection, including RTx, RTx + CTx or RTx + targeted therapies (Supplementary figure 3A-C).

### Characterization of PD-L1 TPS discordance in brain metastasis and extracranial tumor tissue

**Fig. 2:**
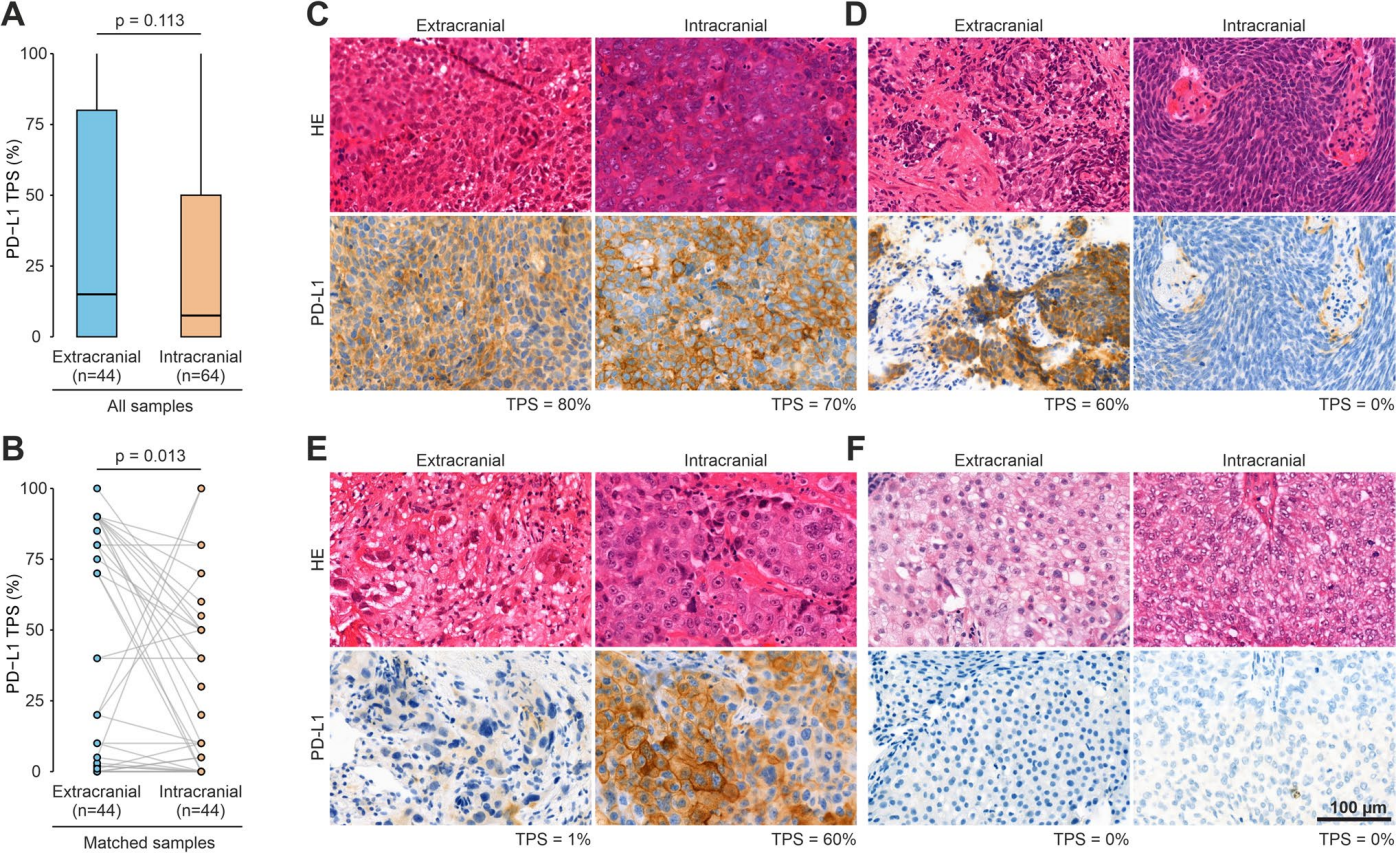
Distribution of intracranial and extracranial PD-L1 TPS. **A** Distribution of PD-L1 TPS in intracranial tissue (*n* = 64) and extracranial tissue (*n* = 44) in the total study cohort (*n* = 64), Mann–Whitney U test statistics. **B** Distribution of matched extracranial and intracranial PD-L1 TPS for those patients, where both extracranial and intracranial PD-L1 TPS was available, paired Wilcoxon signed-rank test statistics. **C-F** Representative HE and PD-L1 IHC images of patients exhibiting (**C**) both extracranial and intracranial high PD-L1 TPS, (**D**) extracranial high PD-L1 TPS and intracranial low PD-L1 TPS, (**E**) extracranial low PD-L1 TPS and intracranial high PD-L1 TPS, and (**F**) both extracranial and intracranial low PD-L1 TPS

The median time between tissue sampling of brain metastasis and extracranial tissue was 15 days (95% CI: 9 – 33). Overall positive rates for intracranial and extracranial PD-L1 TPS (i.e. values ≥ 1%) were 54.7% and 68.2%, respectively (Supplementary table 1). Although we observed a significant correlation between intra- and extracranial PD-L1 TPS (Supplementary figure 3), there was a trend towards higher extracranial PD-L1 TPS with a median of 15.0% compared to a median intracranial PD-L1 TPS of 7.5% (*p* = 0.113) (Fig. 2A, Supplementary table 1).

In 44 patients with available matched intra- and extracranial tissues, we found a significant discordance towards higher extracranial PD-L1 TPS (*p* = 0.013) (Fig. 2B). Exemplary PD-L1 IHC images showing concordant and discordant cases are shown in Fig. 2C-F. Among these 44 patients, median PD-L1 TPS discordance was 9.5% (9.0–95.0, IQR), with 16 patients showing no discordance (35.6%), 29 patients (64.4%) having a discordance of ≥ 1%, and 18 of whom (40.0%) exhibiting a discordance of ≥ 10% (Supplementary table 1).

### Association of intracranial and extracranial PD-L1 TPS with patient outcome

Since we observed discordant PD-L1 TPS in intra- and extracranial tissues, we analyzed the predictive role of both intra- and extracranial PD-L1 TPS in patients treated with RTx + ICI after brain metastasis resection. Results of maximally selected rank statistics, which was used for cut point determination with respect to outcomes of interest, i.e. icPFS, ecPFS or OS, are summarized in Supplementary table 2.

**Fig. 3:**
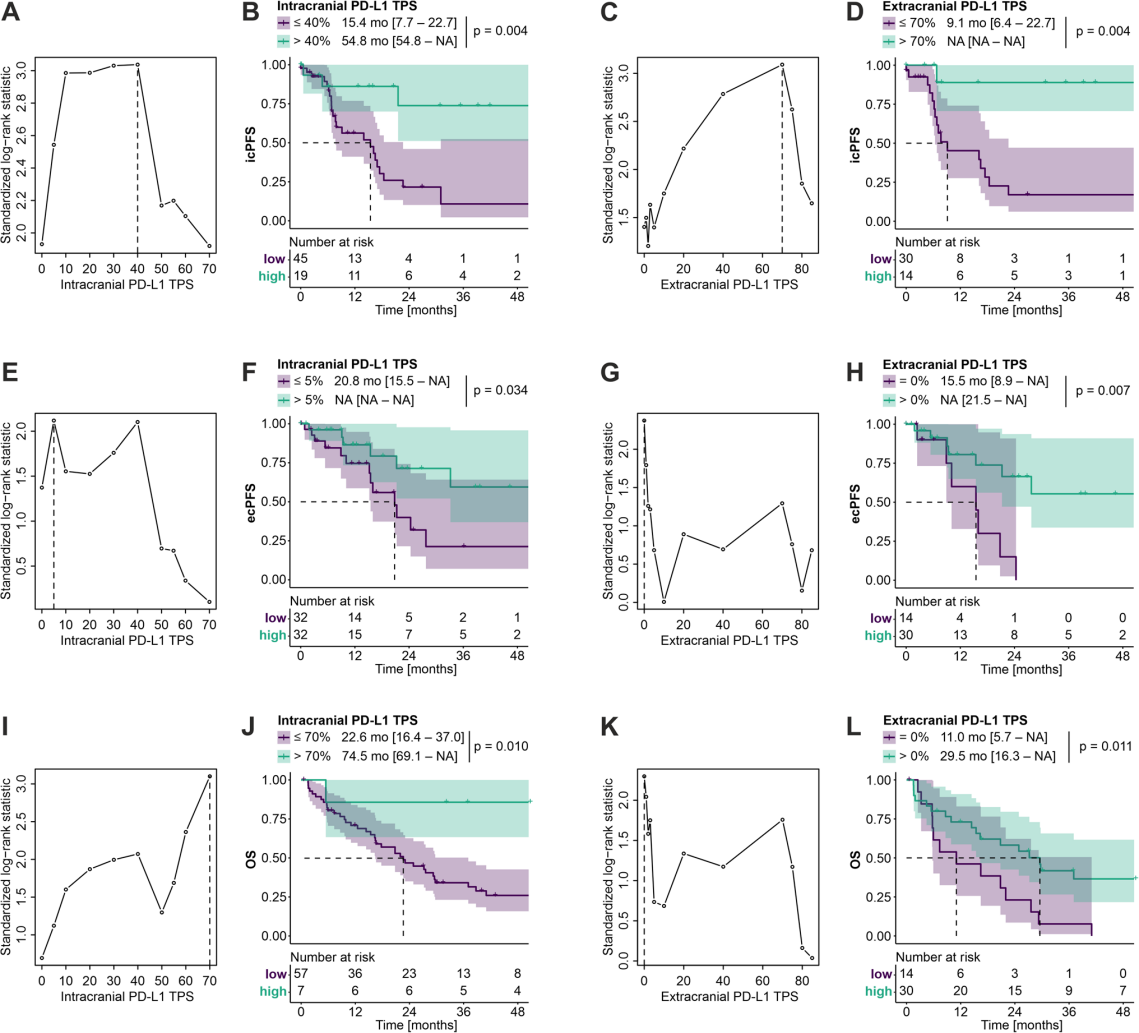
Kaplan–Meier estimates according to intracranial and extracranial PD-L1 TPS. Maximally selected rank statistics and Kaplan–Meier estimates of icPFS, ecPFS, and OS using optimal cut points of intracranial and extracranial PD-L1 TPS, respectively, to dichotomize the study cohort. **A** Optimal cut point determination of intracranial PD-L1 TPS for icPFS, and **B** Kaplan–Meier estimates of icPFS for patients subgrouped accordingly (*n* = 64). **C** Optimal cut point determination of extracranial PD-L1 TPS for icPFS, and **D** Kaplan–Meier estimates of icPFS for patients subgrouped accordingly (*n* = 44). **E** Optimal cut point determination of intracranial PD-L1 TPS for ecPFS, and **F** Kaplan–Meier estimates of ecPFS for patients subgrouped accordingly (*n* = 64). **G** Optimal cut point determination of extracranial PD-L1 TPS for ecPFS, and **H** Kaplan–Meier estimates of ecPFS for patients subgrouped accordingly (*n* = 44). **I** Optimal cut point determination of intracranial PD-L1 TPS for OS, and **J** Kaplan–Meier estimates of OS for patients subgrouped accordingly (*n* = 64). **K** Optimal cut point determination of extracranial PD-L1 TPS for OS, and **L** Kaplan–Meier estimates of OS for patients subgrouped accordingly (*n* = 44)

To predict icPFS, an optimal cut point was identified for intracranial PD-L1 TPS at 40%, while the optimal cut point for extracranial PD-L1 TPS was determined to be 70% (Fig. 3A + C). Patients exhibiting a high intracranial PD-L1 TPS of > 40% vs. ≤ 40% demonstrated a significantly improved median icPFS of 54.8 vs. 15.4 months (*p* = 0.0036) (Fig. 3A-B). Similarly, patients showing a high extracranial PD-L1 TPS of > 70% vs. ≤ 70% had a significantly increased non-estimable median icPFS vs. 9.13 months (*p* = 0.0037) (Fig. 3C-D).

Regarding ecPFS, we identified particularly lower cut points for intracranial and extracranial PD-L1 TPS, being 5% and 0%, respectively (Fig. 3E + G). High intracranial PD-L1 TPS of > 5% vs. ≤ 5% was associated with an increased non-estimable median ecPFS vs. 20.8 months (*p* = 0.034) (Fig. 3E-F). Likewise, patients exhibiting an extracranial PD-L1 TPS of > 0% vs. 0% demonstrated a longer non-estimable median ecPFS vs. 15.5 months (*p* = 0.0068) (Fig. 3G-H).

As for OS, the optimal cut points for intracranial and extracranial PD-L1 TPS were established at 70% and 0%, respectively (Fig. 3I + K). Here, patients with an intracranial PD-L1 TPS of > 70% vs. ≤ 70% had a longer median OS of 74.5 vs. 22.6 months (*p* = 0.0096) (Fig. 3I-J). Similarly, patients exhibiting an extracranial PD-L1 TPS of > 0% vs. 0% showed an increased OS of 29.5 months vs. 11.0 months (*p* = 0.011) (Fig. 3K-L).

Similarly, we investigated the predictive value of PD-L1 TPS discordance between intracranial and extracranial PD-L1 TPS. Here, patients exhibiting a PD-L1 TPS discordance of > 20% vs. ≤ 20% showed a better median icPFS of 54.8 months vs. 16.1 months (*p* = 0.047) (Supplementary figure 3A-B). PD-L1 TPS discordance was not found to be a significant predictor of ecPFS and OS (Supplementary figure 3C-F).

**Fig. 4:**
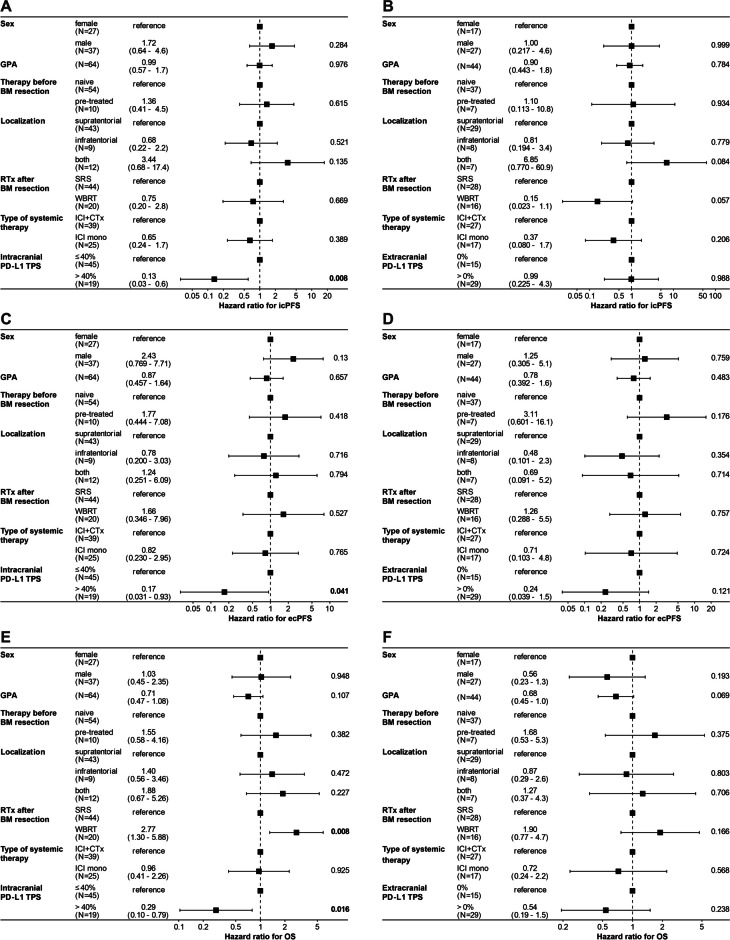
Multivariable Cox regression analysis for icPFS, ecPFS and OS. A + C + E Forest plots reporting hazard ratios and 95% confidence intervals for (**A**) icPFS, (**C**) ecPFS, and (**E**) OS, including patients with available intracranial PD-L1 TPS (*n* = 64), intracranial PD-L1 TPS was dichotomized according to the optimal cut point of intracranial PD-L1 TPS for icPFS (see Fig. 3A). B + D + F Forest plots reporting hazard ratios and 95% confidence intervals for (**B**) icPFS, (**D**) ecPFS, and (**F**) OS, including patients with available extracranial PD-L1 TPS (*n* = 44), extracranial PD-L1 TPS was dichotomized according to the optimal cut point of extracranial PD-L1 TPS for ecPFS (see Fig. 3G). **A-F** Vertical dashed line signifies a hazard ratio of 1.0, p-values indicated, significant p-values highlighted in bold

For multivariable analyses, patients were split into two groups according to the previously determined optimal cut point of intracranial PD-L1 TPS to predict icPFS, i.e. 40% (Table 2). Accordingly, patients with a high intracranial PD-L1 TPS of > 40% had a lower risk of intracranial disease progression (HR 0.13, *p* = 0.008) (Fig. 4A), extracranial disease progression (HR 0.17, *p* = 0.041) (Fig. 4C), as well as a lower risk of death (HR 0.29, *p* = 0.016) (Fig. 4E). The positive predictive value of high intracranial PD-L1 TPS could also be observed in a subgroup of patients who were treatment-naïve before brain metastasis resection for icPFS and OS, but not for ecPFS (Supplementary figure 5A, C, E). The optimal cut point of extracranial PD-L1 TPS to predict ecPFS, i.e. 0%, was not independently associated with patient outcome (Fig. 4B, D, F). In the subgroup of treatment-naïve patients, an extracranial PD-L1 TPS of > 0% was associated with a lower risk of extracranial disease progression (HR 0.09, *p* = 0.034) (Supplementary figure 5B, D, F).

**Table 2:** Patient characteristics in resected NSCLC brain metastasis patients grouped by intracranial PD-L1 TPS cut point of 40%. Summary of patient features of the study cohort (*n* = 64), in which all patients were treated with RTx + ICI after brain metastasis resection. All characteristics are given at the time of first brain metastasis resection (baseline), if not stated otherwise. Patients were grouped by intracranial PD-L1 TPS > 40% vs. ≤ 40%, which was determined to be the optimal cut point to predict icPFS

Characteristics	≤ 40% intracranial PD-L1 TPS, *N* = 45	> 40% intracranial PD-L1 TPS, *N* = 19	*p*-value^1^	q-value^2^
Age, median (IQR)	64 (58 – 72)	64 (58 – 73)	0.95	> 0.99
Sex, *n* (%)			0.095	0.29
Female	22 (49%)	5 (26%)		
Male	23 (51%)	14 (74%)		
Histology, *n* (%)			0.17	0.36
Adenocarcinoma	35 (78%)	19 (100%)		
Pulmonary sarcomatoid carcinoma	1 (2.2%)	0 (0%)		
Squamous cell cancer	7 (16%)	0 (0%)		
Undifferentiated	2 (4.4%)	0 (0%)		
GPA score, *n* (%)			0.080	0.29
Bad GPA score (GPA ≤ 2)	20 (44%)	13 (68%)		
Good GPA score (GPA > 2)	25 (56%)	6 (32%)		
KPS after operation, n (%)			> 0.99	> 0.99
Bad KPS (< 70%)	11 (24%)	5 (26%)		
Good KPS (≥ 70%)	34 (76%)	14 (74%)		
Extracranial metastasis burden, *n* (%)			0.10	0.29
No presence of extracranial metastases	29 (64%)	8 (42%)		
Presence of extracranial metastases	16 (36%)	11 (58%)		
Intracranial metastasis burden, *n* (%)			> 0.99	> 0.99
1 brain metastasis	11 (24%)	4 (21%)		
≥ 2 brain metastases	34 (76%)	15 (79%)		
Tumor volume (mL), median (IQR)	8 (6 – 24)	10 (9 – 14)	0.65	0.89
Edema volume (mL), median (IQR)	87 (59 – 141)	125 (87 – 170)	0.14	0.35
Therapy status before brain metastasis resection, *n* (%)			0.26	0.43
Naive before brain metastasis resection	36 (80%)	18 (95%)		
Pre-treated before brain metastasis resection	9 (20%)	1 (5.3%)		
Primary tumor resection, *n* (%)			0.21	0.39
No primary tumor resection	33 (73%)	16 (84%)		
Primary tumor resection before brain metastasis resection	7 (16%)	0 (0%)		
Primary tumor resection after brain metastasis resection	5 (11%)	3 (16%)		
Mode of adjuvant radiation therapy, *n* (%)			0.071	0.28
SRS	34 (76%)	10 (53%)		
WBRT	11 (24%)	9 (47%)		
Dose of postoperative radiation in Gy, Median (IQR)	48 (0 – 120)	40 (26 – 108)	0.59	0.92
Cumulative pre-operative dexamethasone, median (IQR)	48 (0 – 120)	40 (26 – 108)	0.59	0.89
Cumulative pre-operative dexamethasone dose dichotomized, *n* (%)			0.74	0.92
> 52 mg	21 (47%)	8 (42%)		
≤ 52 mg	24 (53%)	11 (58%)		
Systemic therapy after brain metastasis resection, *n* (%)			0.002	0.026
ICI + CTx (followed by ICI maintenance therapy)	33 (73%)	6 (32%)		
ICI monotherapy	12 (27%)	13 (68%)		
Specific systemic therapy after brain metastasis resection, *n* (%)			0.033	0.25
Carboplatin, Etoposide and Atezolizumab, followed by Atezolizumab maintenance therapy	1 (2.2%)	0 (0%)		
Carboplatin, Nab-Paclitaxel and Atezolizumab, followed by Atezolizumab maintenance therapy	3 (6.7%)	1 (5.3%)		
Carboplatin, Nab-Paclitaxel and Pembrolizumab, followed by Pembrolizumab maintenance therapy	1 (2.2%)	0 (0%)		
Carboplatin, Nab-Paclitaxel, Nivolumab and Ipilimumab, followed by Nivolumab and Ipilimumab maintenance therapy	3 (6.7%)	0 (0%)		
Carboplatin, Nab-Paclitaxel, Pembrolizumab, followed by Pembrolizumab maintenance therapy	3 (6.7%)	0 (0%)		
Carboplatin, Pemetrexed and Pembrolizumab, followed by Pembrolizumab maintenance therapy	14 (31%)	4 (21%)		
Cisplatin, Pemetrexed and Pembrolizumab, followed by Pembrolizumab maintenance therapy	8 (18%)	1 (5.3%)		
Atezolizumab monotherapy	4 (8.9%)	0 (0%)		
Nivolumab monotherapy	1 (2.2%)	1 (5.3%)		
Pembrolizumab monotherapy	7 (16%)	12 (63%)		

^1^Wilcoxon rank sum exact test; Pearson’s Chi-squared test; Fisher’s exact test; Wilcoxon rank sum test

^2^False discovery rate correction for multiple testing

## Discussion

In current clinical routine, PD-L1 TPS serves as predictive biomarker for ICI treatment response, predominantly based on information derived from extracranial tumor tissue. In this observational cohort study, we provide real-world data on the predictive value of brain metastasis-specific intracranial PD-L1 TPS in a cohort of NSCLC patients subjected to brain metastasis resection followed by treatment with ICI.

We observed a significant discordance between PD-L1 TPS of intracranial and matched extracranial tumor tissue. Overall positive rates for intracranial PD-L1 TPS were lower than in matched extracranial tumor tissues, which is concordant to previous studies [15–17]. The higher overall positive rates in our study are likely due to the selection of patients undergoing ICI therapy after brain metastasis resection, while previous studies on intracranial PD-L1 expression were agnostic to systemic treatment modalities.

With respect to clinical outcome, we provide not only information on the role of PD-L1 TPS on overall survival, but also on outcome parameters such as intracranial and extracranial disease progression. Patients with high intracranial PD-L1 TPS, i.e. > 40%, exhibited significantly longer icPFS, ecPFS and OS, compared to those with low intracranial PD-L1 TPS ≤ 40%. These observations underscore the potential utility of intracranial PD-L1 TPS as an independent predictive biomarker for patient outcomes. Specifically in patients with newly diagnosed NSCLC and resected brain metastases, where biopsies of the primary tumor or extracranial metastases are not (yet) available for PD-L1 assessment, intracranial PD-L1 TPS could inform clinicians about patients who would benefit from ICI therapy. It is important to highlight that the optimal cut point for intracranial PD-L1 TPS determined in our study (> 40% vs. ≤ 40%) is virtually similar to the established clinical cut point (≥ 50% vs. < 50%) which qualifies patients for ICI monotherapy [18–20].

We observed that PD-L1 TPS discordance of > 20% was associated with improved icPFS. However, high PD-L1 TPS discordance can be traced back to patients with predominantly high extracranial PD-L1 TPS. Consequently, high extracranial PD-L1 TPS, rather than the discordance itself, is likely to predict icPFS in this setting. As for intracranial PD-L1 TPS, optimal predictive cut points were generally higher compared to extracranial tissue, indicating that higher abundancy of PD-L1 might be required in brain metastases for ICI response. Therefore, dissecting mechanisms inducing a proinflammatory TME, especially in intracranial metastasis sites, could extend the application of ICI in patients with brain metastases [21–24].

Since we analyzed a retrospective patient cohort, our study is limited by heterogeneity in terms of pre-treatment before brain metastasis resection and specific modality and type of ICI treatment after brain metastasis resection. Additionally, we present data comparing brain metastases with extracranial tumor tissue, which were sampled at different time points during the disease of a given patient and without differentiating between different types of extracranial tissue samples (e.g. lymph node biopsy vs. resected or biopsied primary tumor biopsy). Moreover, there is potential bias with regard to the standard diagnostics of PD-L1 TPS as inter-observer variability has been reported for PD-L1 assessment and potential bias due to retrospective collection and storage of tissue with varying storage time, tissue processing and staining cannot be excluded [25, 27].

Prospective, multicenter trials specifically recruiting NSCLC patients with brain metastases are warranted to validate our findings and elucidate the clinical implications.. Future studies should consider immune-modulatory effects of the mutational profile and investigate other immune-related biomarkers as well as interactions within the primary tumor and brain metastasis TME [28–30].

This could in turn could provide a more comprehensive understanding of immunotherapy responses in this challenging patient population. Lastly, digital image analysis could improve PD-L1 assessment by increasing inter-observer reproducibility and should be compared to the results of our study [31].

In conclusion, our study shows that intracranial PD-L1 TPS derived from brain metastasis tissue may inform about outcome, especially intracranial disease progression, and could represent a valuable predictive marker to stratify NSCLC patients with brain metastases for ICI therapy.

### Supplementary Information

Below is the link to the electronic supplementary material.

Supplementary file1 (DOCX 1856 KB)

## Data Availability

No datasets were generated or analysed during the current study.
