# Supplementary material for: Predictive role of intracranial PD-L1 expression in a real-world cohort of NSCLC patients treated with immune checkpoint inhibition following brain metastasis resection
Source: J Neurooncol. 2024 Feb 15;167(1):155–67. doi: 10.1007/s11060-024-04590-w (PMC10978684; doi:10.1007/s11060-024-04590-w)
Supplement: Supplementary file 1 — Supplementary file1 (DOCX 1856 KB) [file 11060_2024_4590_MOESM1_ESM.docx]

Supplementary figures

Predictive role of intracranial PD-L1 expression in a real-world cohort of NSCLC patients treated with immune checkpoint inhibition following brain metastasis resection

David Wasilewski, MD^1, 2, 3^, Julia Onken, MD^1, 2, 3, 4^, Paul Höricke, BSc^5^, Jan Bukatz^1^, Selin Murad^1^, Anton Früh, MD, MSc^1, 4^, Zoe Shaked^1^, Martin Misch, MD^1, 2^, Anja Kühl^4^, PhD, Oliver Klein, PhD^4^, Felix Ehret, MD^6^, David Kaul, MD^6^, Helena Radbruch, MD^7^, David Capper MD^3, 4, 7^, Peter Vajkoczy, MD^1, 2^, David Horst, MD^2, 3, 5^, Nikolaj Frost, MD^2, 8^, Philip Bischoff, MD^3, 4, 5^


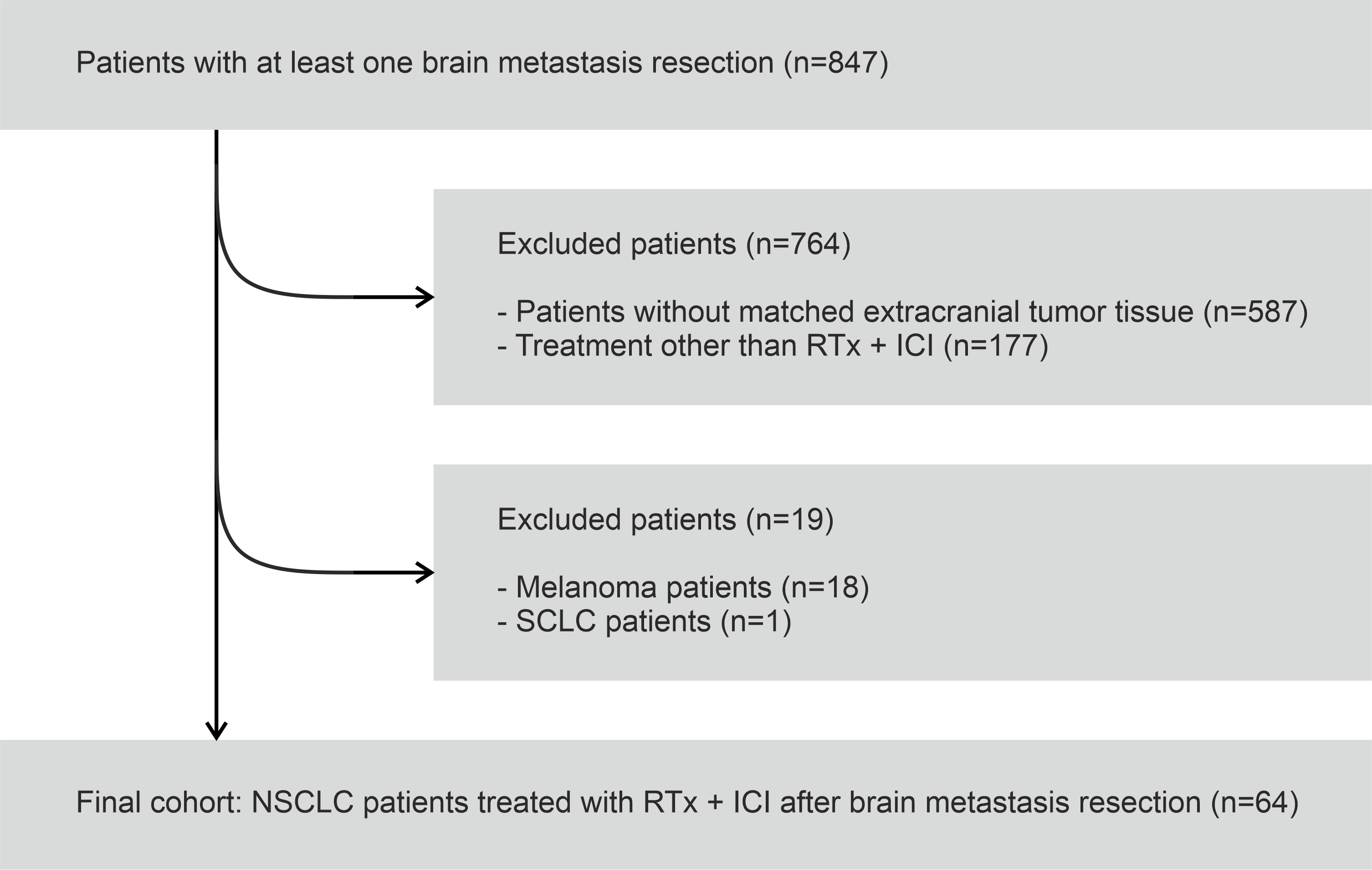


**Supplementary figure 1: CONSORT diagram of included and excluded patients.**

A CONSORT diagram representing the study population with excluded and included patients. Our cohort involved in total 83 patients that underwent brain metastasis resection followed by radiation therapy (RTx) and therapy with immune checkpoint inhibitors (ICIs), from which 19 patients were excluded comprising 18 patients with melanoma brain metastases and one patient with small cell lung cancer (SCLC) brain metastasis. The final cohort included patients with resected non-small cell lung cancer (NSCLC) brain metastases that underwent RTx and systemic therapy with ICIs.


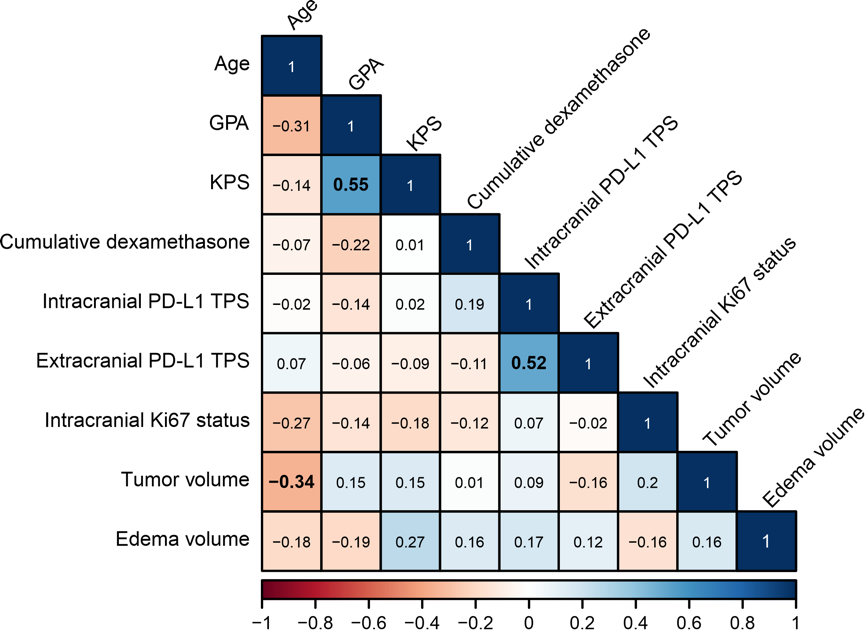


**Supplementary figure 2: Correlation analysis of PD-L1 TPS and clinical covariates**

Correlation between clinical and histopathological covariates within the cohort of NSCLC patients treated with RTx+ICI (n=64), including age, Karnofsky performance score (KPS), gradual prognostic assessment (GPA) score, cumulative pre-operative dexamethasone, intra- and extracranial PD-L1 TPS, intracranial Ki67 index as well as tumor volume and edema volume; statistically significant correlations are highlighted in bold. There was a statistically significant positive correlation between intracranial and extracranial PD-L1 TPS, and between KPS and GPA as well as a statistically significant negative correlation between age and tumor volume.


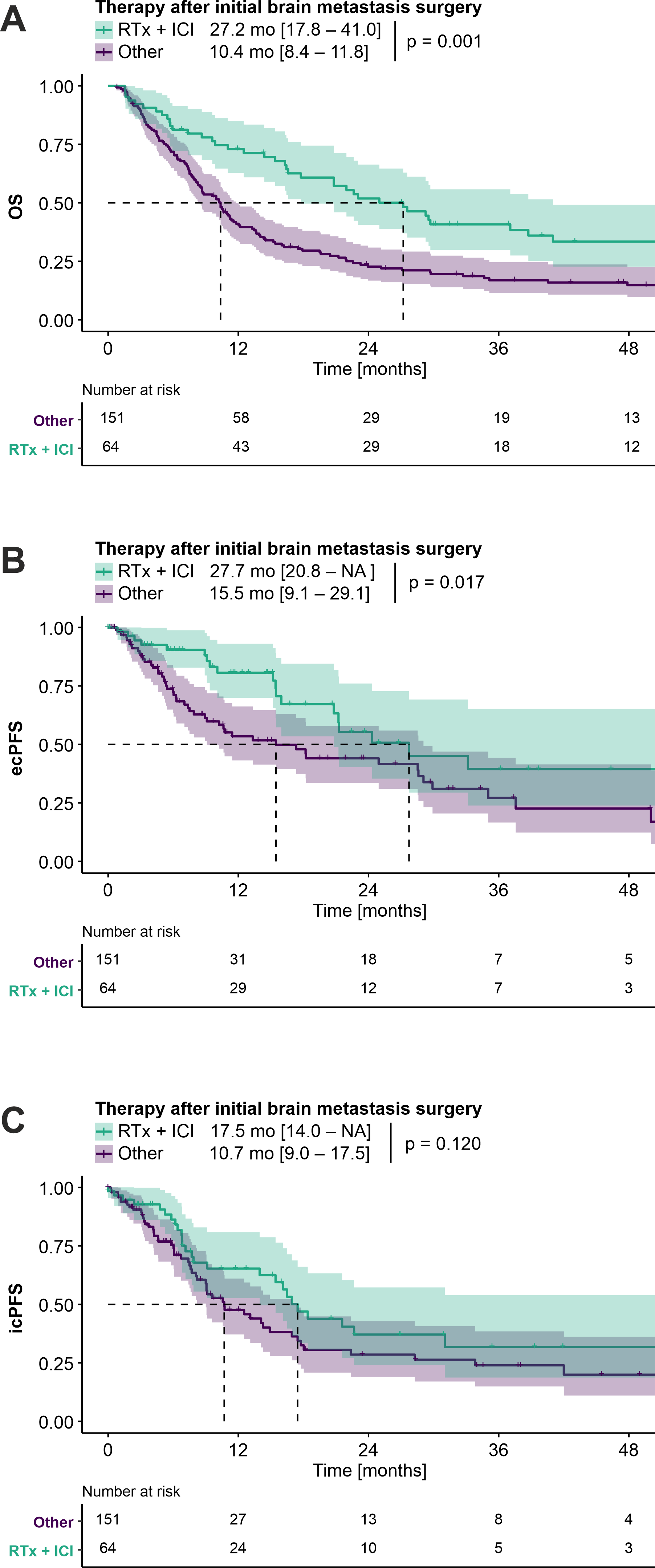


**Supplementary figure 3: Comparison of outcome of ICI-treated vs. non-ICI-treated resected NSCLC brain metastases**

Kaplan-Meier estimates depicting survival outcomes of two distinct adjuvant treatment groups among resected NSCLC brain metastasis patients: 'RTx+ICI' and ‘Other'. NSCLC patients treated with RTx and immune checkpoint inhibition (ICI) (‘RTx+ICI’, n=64) after brain metastasis resection were compared to patients treated with RTx, RTx+chemotherapy or RTx+targeted therapies (‘Other’, n=151) in terms of (**A**) overall survival (OS), (**B**) extracranial progression-free survival (ecPFS) and (**C**) intracranial progression-free survival (icPFS).

| **Characteristic** |  |
| --- | --- |
| **Intracranial tissue (n = 64)** | |
| Overall positivity (intracranial PD-L1 TPS ≥1%) | 54.7% |
| PD-L1 TPS intracranial, median (IQR) | 7.5 (0.0 – 50.0) |
| PD-L1 TPS intracranial, mean (+/-SEM) | 25.5 +/- 4.0 |
| Ki67 intracranial, median (IQR) | 30.0 (20.0 – 50.0) |
| Ki67 intracranial, mean (+/-SEM) | 35.0 +/- 2.7 |
| **Extracranial tissue (n = 44)** | |
| Overall positivity (extracranial PD-L1 TPS ≥1%) | 68.2% |
| PD-L1 TPS extracranial , median (IQR) | 15.0 (0.0 – 100.0) |
| PD-L1 TPS extracranial, mean (+/-SEM) | 36.6 +/- 6.0 |
| Ki67 extracranial, median (IQR) | 40.0 (20.0 – 60.0) |
| Ki67 extracranial, mean (+/-SEM) | 39.4 +/- 3.8 |
| **PD-L1 TPS discordance in matched intra- and extracranial tissue (n = 44)** |  |
| PD-L1 TPS discordance, median (IQR) | 9.5 (9.0 – 95.0) |
| PD-L1 TPS discordance, mean (+/-SEM) | 22.8 +/- 4.3 |
| No discordance between intra- and extracranial tissue | 16 (35.6%) |
| Discordance of ≥1% between intra- and extracranial tissue | 29 (64.4%) |
| Discordance of ≥10 % between intra- and extracranial tissue | 18 (40.0%) |

**Supplementary table 1: Summary of intracranial and extracranial PD-L1 TPS in the study cohort**

PD-L1 TPS values were dichotomized based on a cut point of 1%, i.e. PD-L1 TPS values below 1% were regarded negative. Intracranial PD-L1 TPS was available for 64 patients, extracranial PD-L1 TPS was available for 44 patients. IQR = interquartile range, SEM = standard error of the mean.

| **Predictor variable** | **Outcome parameter** | **Optimal cutpoint** | **Maximally selected rank statistic** | **p value** |
| --- | --- | --- | --- | --- |
| intracranial PD-L1 TPS | icPFS | 40% | 3.0371 | 0.01302 |
| extracranial PD-L1 TPS | icPFS | 70% | 3.0903 | 0.01174 |
| intracranial PD-L1 TPS | ecPFS | 5% | 2.1184 | 0.1432 |
| extracranial PD-L1 TPS | ecPFS | 0% | 2.3745 | 0.0831 |
| intracranial PD-L1 TPS | OS | 70% | 3.101 | 0.0185 |
| extracranial PD-L1 TPS | OS | 0% | 2.2977 | 0.0964 |

**Supplementary table 2: Summary of cut points for intracranial and extracranial PD-L1 TPS**

Summary of the results of optimal cut point determination using maximally selected rank statistics for the numeric predictor variables intracranial PD-L1 TPS and extracranial PD-L1 TPS in relation to outcome parameters of interest, i.e. icPFS, ecPFS and OS.


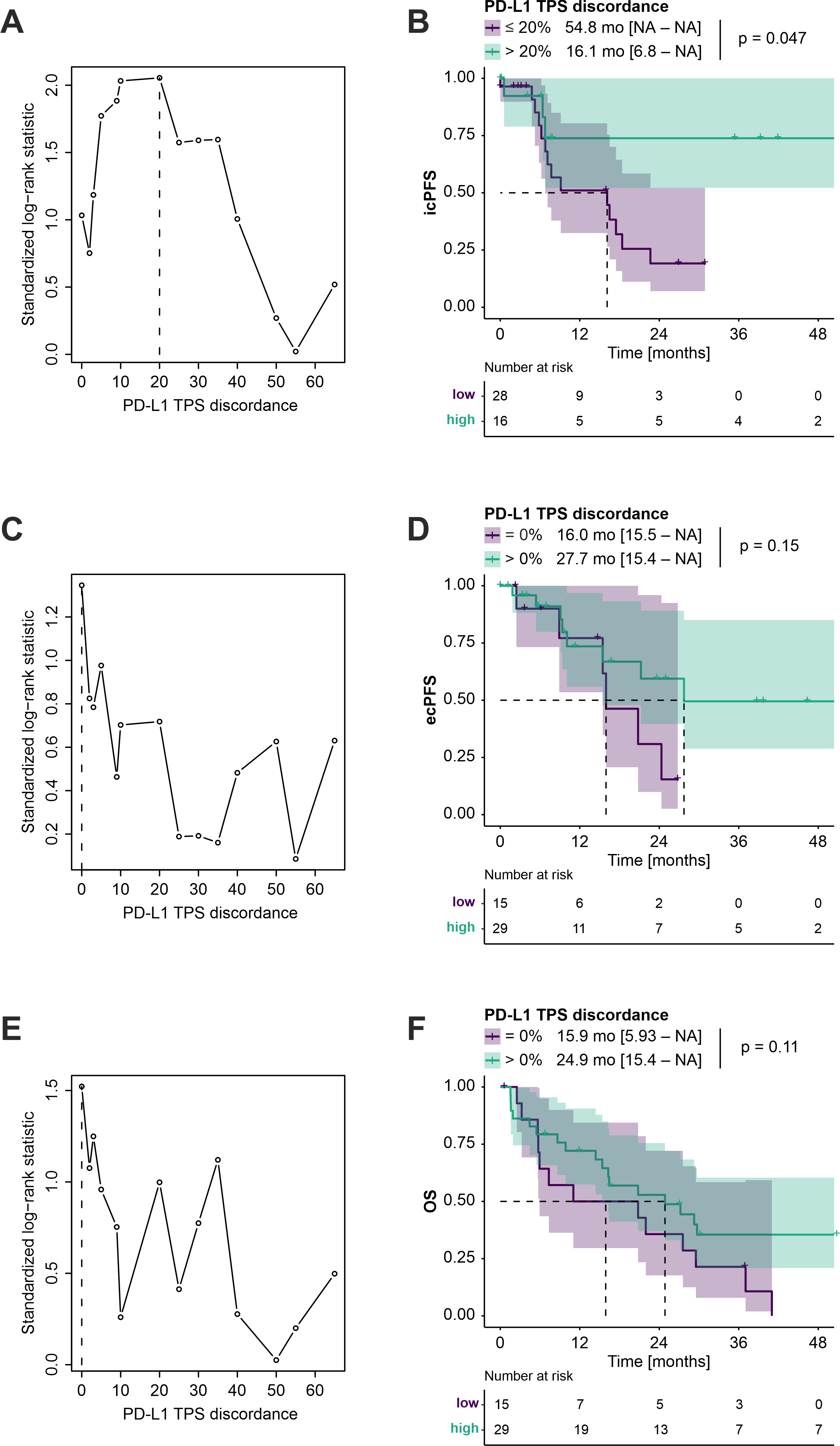


**Supplementary figure 4: Kaplan-Meier estimates according to PD-L1 TPS discordance**

Maximally selected rank statistics and Kaplan-Meier estimates of icPFS, ecPFS, and OS using optimal cut points of PD-L1 TPS discordance to dichotomize the study cohort. (**A**) Optimal cut point determination of PD-L1 TPS discordance for icPFS, and (**B**) Kaplan-Meier estimates of icPFS for patients subgrouped accordingly (n=44). (**C**) Optimal cut point determination of PD-L1 TPS discordance for ecPFS, and (**D**) Kaplan-Meier estimates of ecPFS for patients subgrouped accordingly (n=44). (**E**) Optimal cut point determination of PD-L1 TPS discordance for OS, and (**F**) Kaplan-Meier estimates of OS for patients subgrouped accordingly (n=44).


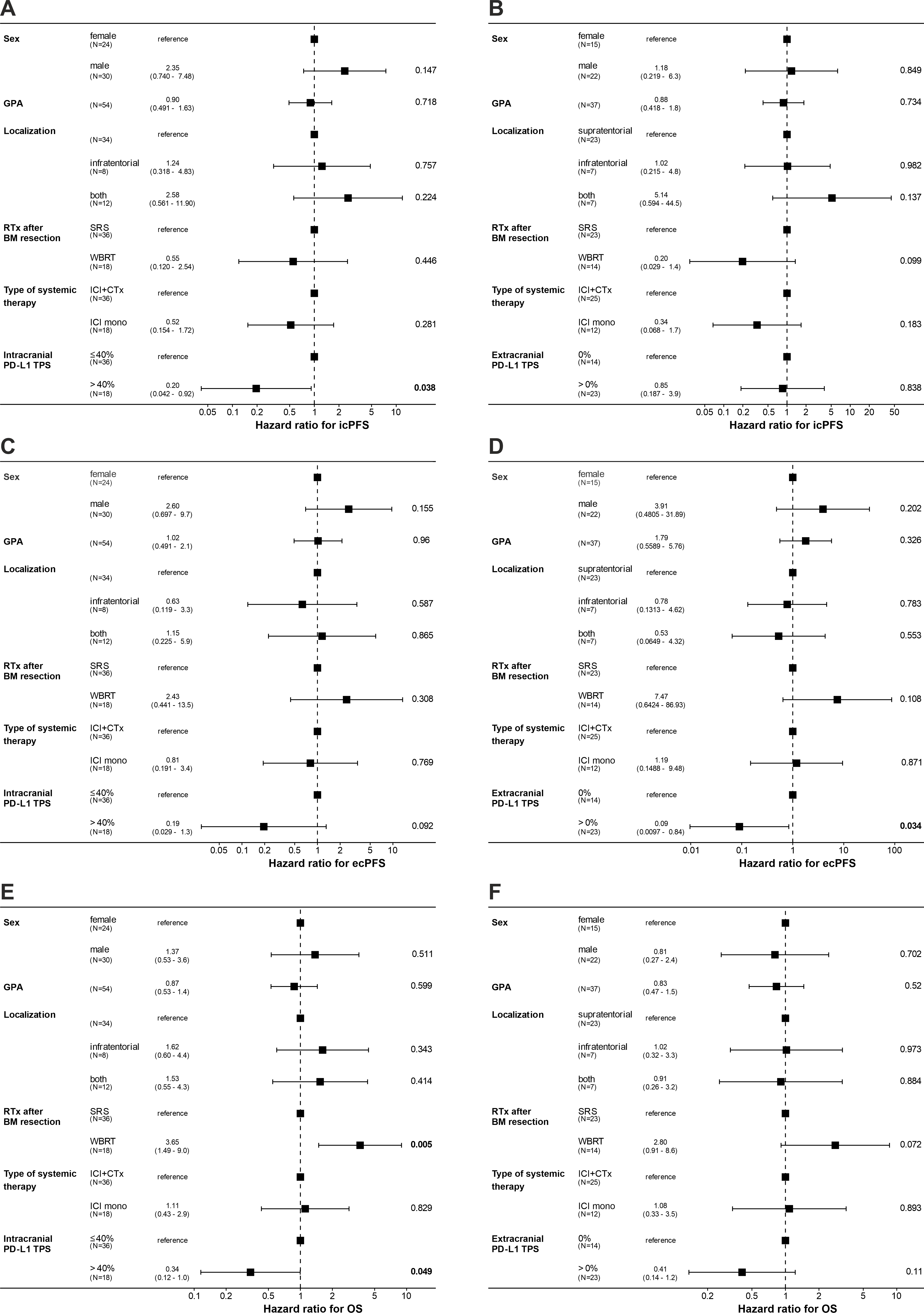


**Supplementary figure 5: Multivariable Cox regression analysis for icPFS, ecPFS and OS in the subgroup of patients without systemic pre-treatment before brain metastasis resection**

In the subgroup of NSCLC patients without systemic pre-treatment before brain metastasis resection (n=54), intra- and extracranial PD-L1 TPS was assessable for 54 and 37 patients, respectively.

(**A+C+E**) Forest plots reporting hazard ratios and 95% confidence intervals for (**A**) icPFS, (**C**) ecPFS, and (**E**) OS. Intracranial PD-L1 TPS was dichotomized according to the optimal cut point of intracranial PD-L1 TPS for icPFS (see Fig. 3A).

(**B+D+F**) Forest plots reporting hazard ratios and 95% confidence intervals for (**B**) icPFS, (**D**) ecPFS, and (**F**) OS, including patients with available extracranial PD-L1 TPS being treatment-naive before brain metastasis resection (n=37), extracranial PD-L1 TPS was dichotomized according to the optimal cut point of extracranial PD-L1 TPS for ecPFS (see Fig. 3G). (**A-F**) Vertical dashed line signifies a hazard ratio of 1.0, p-values indicated, significant p-values highlighted in bold.
